# Continuing professional development in Eswatini: Factors affecting medical practitioners’ participation

**DOI:** 10.4102/safp.v63i1.5395

**Published:** 2021-11-25

**Authors:** Rodney H. Magwenya, Andrew J. Ross

**Affiliations:** 1 Outpatient Department, Mankayane Government Hospital, Mankayane, Eswatini, South Africa; 2 Department Family Medicine, College of Health Sciences, University of KwaZulu-Natal, Durban, South Africa

**Keywords:** continuing medical education, continuing professional development, medical practitioners’ views, motivation, barriers, adult learning theory

## Abstract

**Background:**

The availability of continuing professional development (CPD) activities does not necessarily translate into good participation by health practitioners. Reasons for low participation include time constraints, cost of some activities, irrelevant material and lack of access. This study aimed to explore the views of medical practitioners’ working in Eswatini regarding the factors that affect their participation in CPD programmes.

**Methods:**

A qualitative study using seven in-depth semi-structured interviews and three focus group discussions (FGDs) of medical officers working in the public heath sectors in Eswatini was conducted between November 2020 and February 2021. Open-ended questions were used to explore factors that both motivate and demotivate medical officers participation in CPD activities. The interviews and FGDs were audio-recorded and transcribed verbatim, the qualitative data were analysed using the thematic approach.

**Results:**

The emerging motivating themes described by the participants were: (1) professional responsibility and (2) personal interest and learning need. Whilst the demotivating factors were: (1) non-relevance to clinical practice, (2) cost of participation, (3) lack of reward, and (4) no recognition for staying up-to-date.

**Conclusion:**

The motivating factors are associated with deep learning and linked well with the principles of adult learning. The demotivating factors found were in keeping with findings from other studies in a variety of countries. It is important for the Medical Council and CPD organisers to be aware of the different motivations and de-motivations for practitioners to engage in CPD to enable them to plan and implement their programmes effectively.

## Introduction

Continuing professional development (CPD) is the purposeful updating and improving of professional knowledge and competence that is generally undertaken throughout an individual’s working life.^1^ It is regarded as essential for healthcare professionals (HCP) to enable them to improve their knowledge and clinical care practices.^2,3^ However, compliance with CPD is a major concern in many countries and making it mandatory may not be as effective as addressing the educational quality of the activities offered.^4,5^ It is therefore important to understand what motivates HCP to participate in CPD to improve compliance.

Based on the literature, there are five broad categories of motivations as to why medical practitioners participate in CPD activities.^6,7,8,9,10,11^ These include: (1) the expectation to be knowledgeable and up-to-date, (2) a topic of interest, (3) to improve behaviour or skills learned, (4) to learn more about a specific condition, and (5) the need for social contact with other practitioners and to escape from the daily work routines.^6,7,8,9,10,11^ Irrespective of the motivating factor, for learning to be effective it must be based on sound educational principles.

Many theories have been proposed to describe and conceptualise learning in the medical profession in order to apply these educational principles to develop CPD activities. One such theory is andragogy (adult leaning theory), which refers to the methods used by adults to learn. It has long been recognised that the learning process for adults is different from children, and it varies amongst them in terms of needs and styles.^12,13^

Knowles highlighted five important assumptions about the way adults learn^12^; these include the importance of self-directed learning, their ability to draw from previous experiences in order to learn new things and a recognition that they learn more easily when they are able to identify a reason for acquiring new knowledge or skill. In addition, adults want to learn things that are applicable to their practice and finding the motivation to learn is important for improving self-esteem and career advancement.^12,13,14^

In most countries, including Eswatini, attendance at formal CPD activities largely remains poor, with opportunities not founded on adult learning theories cited as one of the reasons for this.^2,7,15^ Other reasons include a lack of accessibility of activities (especially in more remote and rural regions), irrelevant topics, time constraints (for already overworked and busy professionals), and lack of personal motivation.^4,7,10^ Whilst the goal of CPD is behaviour change and improved clinical practice, there is some controversy as to how and whether this can be achieved through CPD programmes. Ideally, HCPs should be self-directed learners who are able to take charge of their own learning by identifying their individual learning needs, setting goals and using appropriate resources and activities to attain them (applying knowledge to practice) rather than forcing participation in compulsory CPD activities.^16^ Various strategies for CPD have been developed globally to encourage self-directed learning (e.g. experiential and reflective, portfolio, problem-based and discovery-based learning).^16^ Self-directed learning is regarded as an efficacious method of CPD, with individual motivation being a key factor in ensuring its success.^17^

Understanding the motivation of doctors in Eswatini to attend CPD activities is important for creating relevant programmes, improving participation, and hopefully encouraging a change in behaviour and practice. Gibbs classified learning into surface and deep learning.^18^ He described knowledge acquisition as surface or ‘superficial’ learning, which is not sufficient for the development of critical thinking or application of gained knowledge to practice.^18^ Such learning is motivated by an external factor such as an examination or in the context of CPD for HCPs, the need to acquire points.^18^ Deep learning on the other hand goes beyond knowledge acquisition and seeks to understand the underlying principles and being able to apply those principles in practice.^18^ Motivating practitioners through CPD points alone is unlikely to lead to sustainable deep learning as described by Gibbs, because it stems from an external source.^18^ In addition, research has shown that when adult learners feel that their personal development is valued and indeed expected, they are more likely to engage in deep learning and to apply knowledge gained into practice.^18,19^ Internal motivation and collaborative work are regarded as some of the pillars of deep learning, with issues such as satisfaction of understanding new concepts and joy of finding applicability of knowledge being likely to be more sustainable in the long-term than external ones (financial reward or CPD points).^18^

Whilst the Eswatini Medical and Dental Council (EMDC) views CPD as important, there is currently no framework in the country and no guidelines for practitioners to know what constitutes acceptable outcomes. Under the current system, medical practitioners are informally encouraged to take part in CPD programmes on an ad hoc basis that are mainly provided through non-governmental organisations (NGOs) and the Ministry of Health. In addition, they are encouraged to participate in online CPD and to establish in-service committees at the hospitals where they work, with these committees being responsible for journal club meetings and CPD activities. The EMDC is in the process of formulating a formal CPD system. Although there is considerable literature from around the world about CPD programmes and how they are structured, there is a paucity of research in this area in Eswatini that might inform the EMDC as they develop this system, especially regarding the factors that motivate practitioners to engage in CPD. In developing a new system, it is important for the EMDC to consider the views of practitioners to ensure that they have ownership of their CPD.^14,20^ One way to do this is to address the issues that drive local practitioners to participate in CPD and identify how these considerations can be incorporated into the system being developed. This study aimed to establish the opinions of practitioners working at government healthcare facilities in Eswatini regarding factors affecting their engagement in CPD activities. It also aimed to make recommendations about how CPD could be implanted in the country.

## Methods

This qualitative study was conducted between November 2020 and February 2021 using in-depth semi-structured interviews and focus group discussions (FGDs) of medical practitioners working in Eswatini public sector hospitals. The FGDs were based on an interview and guide that was developed using the results from an earlier scoping review. The use of these two methods of data collection enabled data triangulation, which enhanced the data richness and depth of inquiry and increased the validity of the findings.^21^

A systematic but purposeful sampling technique was used to identify public sector medical practitioners registered with the EMDC who were invited to participate in the study. From the list of 297 practitioners registered with the EMDC who had correct and updated contact details, every 29th practitioner was selected for inclusion. Out of 25 participants who were approached to participate in the study, 17 agreed, with the remaining who declined citing their busy schedules and the coronavirus disease 2019 (COVID-19) pandemic amongst the reasons for their decision.

All potential interviewees were informed telephonically about the study’s aims, what their participation entailed, its voluntary nature and the steps taken to ensure their anonymity and data confidentiality. They were also provided with information to help them reach an informed decision before agreeing to participate and signing the informed consent forms. Coronavirus disease 2019 protocols were followed in compliance with the Ministry of Health guidance for both data collection methods.

Individuals who were able to meet at the selected locations were assigned to the focus group in that region, whilst those who were unable to meet participated in in-depth interviews. Seven face-to-face interviews and three FGDs were conducted by the first author, each lasting from 30 min to an hour. The three FGDs were conducted with 10 participants from the three administrative regions of Manzini (four participants), Hhohho (three participants) and Shiselweni (three participants), although the participant numbers were relatively small they managed to engage in rich discussions.^22^ An open-ended question: ‘What motivates you to participate in CPD programmes?’ was used to initiate the discussion in both the interviews and the FGDs. Summarising their comments and asking for further explanations were used as prompts to provide clarity about what the participants were saying. An interview and a FGD were carried out, then the data were reviewed and analysed for each participant before moving on to the next session. Data saturation was reached when no new themes emerged.

The FGD and interview audio recordings were transcribed verbatim and the data were managed using NVIVO^®^.^23^ The data were analysed via constant comparison analysis, which allowed the assessment of saturation in general, as well as across-group saturation. Data saturation was reached when no new themes were identified from the final FGD. Two researchers read the transcripts repeatedly to become familiar with the data, after which they developed a coding framework and coded the transcripts separately. Line by line coding was used to form free codes that were then grouped to form themes and later combined to make categories. The coding framework developed was applied to all transcripts and any new codes or themes that emerged. When the two researchers identified different codes, these were discussed until consensus was reached on the final list of codes and themes.

### Ethical considerations

The required ethical approval was obtained from the National Health Research Review Board (Eswatini) and the Biomedical Research Ethics Committee of the University of KwaZulu-Natal (BREC/00001700/2020), South Africa.

## Results

A total of 11 men and six women participated in the study, their ages ranging from 25 to 67 years, with the duration of their practice ranging from 1 to 35 years. All were medical doctors working in public sector hospitals, with two being specialists and the rest general practitioners (GPs). The factors affecting their participation in CPD activities were divided into those that motivate and demotivate them to attend.

## Motivating factors

Two main themes emerged from the data analysis regarding the motivating factors, these being professional responsibility and personal interest and learning needs.

### Theme 1: Professional responsibility

The most common acknowledgement was that attending CPD activities was part of their professional responsibility to engage in various forms of acquiring new knowledge and skills, this being regarded as an important motivation for the participants:

‘It goes without saying that CPD is a fundamental activity for all practitioners; the question may be how to go about it though.’ (Female, GP, 57 years old)

There was general agreement that healthcare practitioners needed to take part in CPD activities as part of fulfilling the life-long learning obligation they made when they joined the profession. Two sub-categories were observed in the data when looking at the participants’ knowledge about CPD in Eswatini. The first was the ‘aware participants’, who felt an obligation to engage in CPD because of where they practised before coming to Eswatini, whilst recognising that there was no formal enforcement of CPD points in the country:

‘Where I used to practice, they required us to accumulate a certain number of points for us to be able to renew our licences; luckily, here it’s not yet like that. But I still wish to keep up that practice of doing my CPD [*continuing professional development*].’ (Male, GP, 39 years old)

The second sub-category was ‘unaware participants’, which consisted of those who were not aware of the current CPD system in Eswatini, but participated as they were either from countries where it was compulsory to do CPD or had heard in medical school that it is an important and necessary part of being a doctor:

‘It’s unclear how many points one should accumulate to be able to be eligible for re-registration or to be compliant with the requirements, so I just do as much as I can just in case.’ (Male, GP, 27 years old)

‘I’m really not sure what points are required per year, but I know even from my medical school years that we have to do CPD [*continuing professional development*].’ (Female, GP, 25 years old)

### Theme 2: Personal interest and learning need

Another common motivation for engaging and completing CPD activities was the issue of personal interest of a professional nature. As they all worked in public sector facilities, they were also likely to manage health conditions that are particular to the country and want to keep abreast of developments in the field:

‘Let’s have more opportunities to do CPD [c*ontinuing professional development*] which is line with our interests. We would definitely attend these.’ (Male, GP, 27 years old)

Some felt that their decision to take part in CPD was motivated by a clinical case they were dealing with (or had dealt with) and wanted to learn more about managing such cases:

‘I commonly get to know what I don’t know after reviewing an interesting or difficult case. Then I try and read around the topic from trusted medical websites like Medscape[^®^]. The guidelines available I feel do not answer most of my questions; though I am hopeful the new and upcoming guidelines will tackle some of the shortcomings of previous ones.’ (Male, GP, 37 years old)

The emergence of a new disease (COVID-19) also featured prominently as a motivation for engaging in CPD, and whilst they wanted to know more in general about the virus to provide appropriate patient care, they also wanted more information to protect their loved ones. Others also mentioned the issue of misinformation on social media as a driver for them to attend formal CPD on COVID-19:

‘I rarely saw the usefulness of CPD [*continuing professional development*] until we had COVID [*coronavirus disease*] in the country; it was a new disease with lots of conflicting messages in the media. I had to go for special CPD courses organised by the Ministry to learn how to protect myself, my family and also help my patients.’ (Male, GP, 39 years old)

## Demotivating factors

Four themes emerged under demotivating factors, these being non-relevance to clinical practice, the cost participating in CPD activities (time and finance), lack of reward, as well as no recognition of achievements.

### Theme1: Non-relevance to clinical practice

A number of participants stated that they would not attend CPD activities that were not relevant to their practice, as this was a waste of their time and did not contribute to improving patient management:

‘Most CPD [*continuing professional development*] activities are less practice oriented. They don’t apply to where we are. We can learn some skills but if we can’t apply them to our practise they are useless to me, and I therefore won’t attend.’ (Male, GP, 37 years old)

Others were concerned that they were not consulted in identifying CPD activities and topics, given that they worked in under-resourced public sector facilities and were well equipped to know what they needed to learn about:

‘Local experts and practitioners should be heavily involved in designing and coming up with content, as they are aware of some of the challenges unique to our setting that an outsider may not really appreciate.’ (Female, GP, 31 years old)

### Theme 2: Cost of participation

The expense and time constraints associated with attending some CPD activities were stated as a disincentive by some practitioners, as they not only had to pay for the courses but also for the travel and accommodation costs. Their high patient numbers and lack of staff to replace them should they be away made it difficult for them to leave their posts:

‘There are some international and regional CPD [c*ontinuing professional development*] meetings that we fail to attend due to lack of sponsorship; they are too expensive for us to even consider paying for them as individuals. So, we may miss out on discussing new trends with our peers.’ (Male, Specialist, 65 years old)

‘Many practitioners already feel overworked and on the brink of burnout if not already there, with most hospital units stretched very thin, there is hardly enough time for CPD [c*ontinuing professional development*] unless some major changes happen.’ (Male, GP, 39 years old)

### Theme 3: Lack of reward

There was a recurring sentiment amongst the participants that they did not want to participate in CPD activities if this was not linked to some career reward or financial compensation. There was an expectation that engaging in CPD should lead to better career prospects and therefore improved remuneration. The only other means of improving their career options was to study for a higher qualification, which they did not have the time or the inclination to do. The statements reflect the view that engaging in CPD did not translate to career advancement, and that irrespective of how much CPD was performed, it would not change their status at their place of employment:

‘… Other small courses should be recognised and lead to better compensation or career prospects, which is not the case currently.’ (Female, GP, 41 years old)

‘In the past, when you attended a CPD [c*ontinuing professional development*] meeting, you were reimbursed for the fuel used, and at times even given some stipend, therefore attendance was always very good.’ (Female, GP, 57 years old)

Some expected a financial incentive to participate, such as tax deductions, as in settings such as Malawi:

‘I practised in Malawi for a while, and what I liked about CPD [c*ontinuing professional development*] there was that in the event you paid for some event, this would be considered as a tax-deductible expense.’ (Female, GP, 41 years old)

### Theme 4: No recognition for staying up-to-date

There was also a feeling amongst the participants that their acquisition of new knowledge and skills obtained at CPD courses were not appreciated by those in authority, which they contended was a reason for the low turnout. Whilst they may want to upskill themselves, the absence of the recognition for keeping abreast with developments in their field made them feel that their employer did not care if they stayed up to date. In the absence of a formal CPD system with points being awarded for qualifications, they were concerned that their own private efforts to improve their knowledge and skills would not be acknowledged:

‘The issue of self-development needs to be tied to a system of recognition of such endeavours. Otherwise the motivation to self-develop is not there.’ (Male, specialist, 65 years old)

‘The current system doesn’t allow us to grow … When we look at private learning and qualifications obtained will these be considered for CPD [c*ontinuing professional development*] points in the system?’ (Male, GP, 42 years old)

## Discussion

The emerging motivating themes described by the participants were: (1) professional responsibility and (2) personal interest and learning need; whilst the demotivating ones were: (1) non-relevance to clinical practice, (2) cost of participation, (3) lack of reward, and (4) no recognition for staying up-to-date.

This study found that professional responsibility was an important motivating factor for participating in CPD activities for most practitioners. This aligns well with the literature regarding the motivation behind doctors’ decisions to engage in CPD; the issue of professional duty being fundamental to their participation as articulated by Cervero and Richards and Cohen.^6,8^ Most participants agreed that there was an expectation for any practising doctor to engage in CPD to stay abreast of their field. By putting in place a clearly outlined system with mandatory and enforceable CPD point target, the EMDC can fulfil its mandate of regulating the profession and ensuring that quality care is given to patients, as required by the Medical and Dental Practitioners Act of 1970 and encourage practitioners to participate in CPD.^10,24^ However, this must be communicated effectively to remove any doubts for both ‘aware’ and ‘unaware’ professionals, as one of the recognised causes of noncompliance with CPD activities in other parts of the world is poor communication and lack of comprehension of their importance and requirements.^20^

With regard to personal interest and learning needs, participants expressed a motivation to participate in a CPD activity that would answer particular clinical questions they might have which is in keeping with the literature on participation in such activities.^8,9^ This intrinsic motivation for learning should lead to greater self-exploration, discovery and reflection, all of which are important components of deep learning. Some of the CPD activities in Eswatini are hospital based (such as journal clubs/teaching ward rounds), making it more practice-based than those run by pharmaceutical companies, which are usually related to their products.^25^ The EMDC needs to incorporate hospital-based CPD activities by setting standards for them to be acceptable for consideration and indicating the number of points that can be awarded. Many CPD activities continue to be performed by NGOs and other partners, which may not be in line with the interests of practitioners. The EMDC needs to put mechanisms in place for providers to carry out needs assessments before implementing their activities and make it mandatory for participants to evaluate the CPD activities they attend.^26^ The evidence suggests that learning through CPD activities may lead to changes in practice, if a needs assessment is carried out and what is learnt is linked to practice.^26,27^

Non-relevance to clinical practice was found to be one of the demotivating themes emerging from the data, with a study in Iran finding that impractical CPD activities were a major concern of practising doctors.^7^ This was supported by a study in Denmark, which concluded that what was learnt had to be relevant for it to have an impact.^28^ In this study, some participants suggested that local specialists and experts needed to play a larger role in identifying CPD content and be involved in facilitating the events. This is an important consideration which was supported by Peloso and Stakiw, who found that CPD was more likely to be effective in leading to improved practice if information was presented by trusted peers or local experts.^29^ The EMDC should also consider giving guidelines for CPD providers that make it necessary for the content to be evaluated by local experts and for their inclusion in conducting the events. Such capacity building and clinical governance needs to become a cornerstone of a sustainable CPD system.^29^

A recurring theme amongst participants was the cost of participation, both in terms of financial and time, as reported by a survey of doctors in Spain, which also identified these two issues as important determinants for physicians’ CPD attendance.^30^ This is similar to the findings of a national survey in the United States of America, which found time to be one of the most important barriers to attending CPD across specialities.^31^ In an attempt to deal with the issue of time constraints, the United Kingdom provides ‘protected time’, which enables practitioners to use time during work hours to attend some of their CPD activities.^32^ and was supported in a Spanish survey also.^30^ In Eswatini, protected time is generally not available, except for programmes run by the Ministry of Health and its partners that require travel to a distant city to attend. To mitigate the issue of cost to participate (mainly because of cost of travel), remote learning opportunities could be made more accessible, as has been the case for the COVID-19-related CPD events. In addition, in developing compulsory CPD in Eswatini, consideration needs to be given to how time can be made available for medical officers to participate in CPD activities and be included in the general training plans for medical officers. Such a protected time system would require their clinical responsibilities during that period to be covered by their peers and senior colleagues.^4^

The lack of reward emerged as a theme in the data that is in keeping with the need to improve their financial position or status by engaging in CPD activities.^8,9^ However, if participation is based solely on financial reward as a motivation, learning is likely to be superficial, where practitioners do not go beyond the acquisition of knowledge and may make no attempt to understand facts or put then into practice.^18^ When developing a compulsory CPD programme in Eswatini, addressing both the incentives and the disincentives to participate are important. Although the financial incentives are seen as resulting in only superficial learning, addressing this aspect can remove a major disincentive to participate in CPD activities. In Malawi and the United Kingdom (for self-employed doctors), CPD costs are tax deductible.^33^ An option would be for the EMDC to engage government and motivate for a tax incentive system for those who wish to take part in paid outcomes-based CPD events.

There was some despondence amongst participants who felt there was no recognition of their CPD achievements by authorities. According to Gibbs, when personal development is valued and expected, learners (in this case medical practitioners) will be more likely to apply the knowledge gained and exhibit more critical and analytical thinking,^18^ which are all attributes of deep thinking and learning. In dealing with this issue, the EMDC could consider having a reciprocal recognition system with its neighbouring countries, which means that CPD activities performed via recognised providers within the region and other selected jurisdictions could be submitted for local credits. Such recognition mechanisms are already in place in Namibia, where CPD points from South Africa are recognised.^34^ It will also be important for the EMDC to set out guidelines on all activities acceptable for CPD credit and to include the number of points that can be claimed for attending certain courses (short courses, diploma courses and longer programmes). In addition, to encourage participation in meaningful CPD programmes, the Ministry of Health could consider putting in place a career notch progression system that would recognise a practitioner’s experience and link it to their outcomes-based CPD. Outcomes-based CPD would consider the impact of learning activities on both change of practice by participants and patient outcomes.^35^ This may take a similar form to the Chinese CPD system, in which the credits are linked to annual performance appraisals, credentialing and promotions.^36^

Only one of the five motivations described in the literature was not seen in this study,^6,8^ this being related to meeting and interacting with colleagues or escaping from daily routines. It is unclear whether this was related to changes in how CPD activities were performed during the COVID-19 pandemic, with most being performed via online platforms, such as Zoom^®^.

The four principles of adult learning by Knowles are applicable to the findings from this study.^12^ Firstly, it is important to recognise that adults need to be involved in the planning and evaluation of their learning activities to ensure that CPD activities are self-directed and learner-driven.^9,12^ The sentiment amongst the practitioners of personal interests not being well addressed by current activities supports this assertion and that they are not involved in topic identification as those covered were not perceived to be relevant to their practice. Secondly, experience provides an important basis for learning, this being evident when participants expressed a need to improve their patient management when dealing with a similar case as a motivation for attending CPD activities. Thirdly, adults prefer learning activities that are relevant to them and impact their work and this is indicated by the need for CPD activities that address their interests and are applicable to their work. Fourthly, adult learning should be problem centred rather than merely didactic or content-oriented, which aligns well with one of the motivations expressed by practitioners that their learning needs should be based around their working environment. It is important that adult learning theory is incorporated into any CPD programme, as it addresses intrinsic motivation to learn, lifelong learning and the application of learning to practice.

The findings from this study need to be considered with an understanding of its limitations. It is a qualitative inquiry with a small sample size and the results may therefore not be generalisable beyond the participants, although they may give insights as to what may motivate a similar cohort of medical practitioners. The small participant numbers in the focus groups is a limitation. However, according to Fern more information may be obtained in conducting two groups of a smaller participant number (four) than a single group (of eight).^37^ In the case of this study, we looked at conducting our three focus groups with smaller participant numbers rather than having one with 10 participants. This was also influenced by the COVID-19 pandemic and the desire to ensure participant’s safety. The practitioners were all based at Government hospitals and their views may be different from those working in private settings.

## Conclusion

Eswatini medical practitioners engage in CPD for a variety of reasons, amongst these, two are linked to deep learning, namely (1) to address a particular learning need and (2) satisfaction of having their achievements recognised. It is important that in planning and implementation of CPD activities, organisers consider these motivations and ways in which adults learn. Future research should focus on the preferred methods of achieving effective CPD (in terms of patient outcomes and change of practice).
